# Clinical Features of Iris Cysts in Long-Term Follow-Up

**DOI:** 10.3390/jcm10020189

**Published:** 2021-01-07

**Authors:** Joanna Konopińska, Łukasz Lisowski, Zofia Mariak, Iwona Obuchowska

**Affiliations:** Department of Ophthalmology, Medical University of Białystok, Jana Kilińskiego 1 STR, 15-089 Białystok, Poland; lisowski@vp.pl (Ł.L.); mariakzo@umb.edu.pl (Z.M.); iwonaobu@wp.pl (I.O.)

**Keywords:** ultrasound biomicroscopy, biometric measurement, anterior segment cysts, iris cysts

## Abstract

This study evaluated the characteristics and clinical course of patients with iris cysts in the long-term follow-up (24–48 months). We retrospectively analyzed the medical records of 39 patients with iris cysts (27 women and 12 men). Age, visual acuity, intraocular pressure (IOP), slit-lamp evaluation, and ultrasound biomicroscopy images were assessed. The mean age at diagnosis was 40.6 ± 17.48 years. Thirty (76.9%) cysts were peripheral, five (12.8%) were located at the pupillary margin, two (5.1%) were midzonal, and two (5.1%) were multichamber cysts extending from the periphery to the pupillary margin. A total of 23 (59%) cysts were in the lower temporal quadrant, 11 (28.2%) were in the lower nasal quadrant, and 5 (12.8%) were in the upper nasal quadrant. Cyst size was positively correlated with patient age (rs = 0.38, *p* = 0.003) and negatively correlated with visual acuity (rs = −0.42, *p* = 0.014). Cyst growth was not observed. The only complication was an increase in IOP in three (7.7%) patients with multiple cysts. The anatomical location of the cysts cannot differentiate them from solid tumors. The vast majority of cysts are asymptomatic, do not increase in size, and do not require treatment during long-term follow-up.

## 1. Introduction

The elevation of the iris seen on a slit lamp examination is always of concern to the ophthalmologist because of the suspicion of a tumor in the iris or ciliary body. However, in 76% of cases these are benign changes, most often cysts (38%) [1]. Policies developed by Shields et al. [2] are intended to help distinguish benign changes such as iris cysts from melanoma. The high probability of a malignant lesion is indicated by such morphological features as the location of the lesion in the lower quadrants of the iris, ectropion and/or diffuse nature of the iris elevation.

The cyst classification proposed by Shields et al. [3] introduced a division into primary and secondary cysts, depending on their tissue origin. Primary cysts, which are epithelial in origin, predominate. They have thin, regular walls and a hypoechoic interior. Their sizes usually do not exceed 3 mm. Primary cysts rarely cause complications or impair visual acuity [3]. Secondary cysts may result from implantation of the conjunctival epithelium, cornea, or eyelid skin into the iris, tumor metastasis, parasitic infections, or chronic use of miotics [4]. The cause of the formation of secondary cysts is most often trauma to the penetrating eyeball or surgical intervention. They can take the form of compact masses (“pearls”), reservoirs filled with fluid (serous cysts), or cause intraepithelial growth. They usually have large dimensions (approximately 5 mm in cross-section) and thick walls (approximately 0.4 mm). Their growth is varied; initially they can increase rapidly and remain the same in the later period.

Secondary cysts may reach a significant size and lead to the development of numerous complications, such as corneal edema, uveitis, secondary closure-angle glaucoma, astigmatism or cataracts caused by pressure on the lens, and iris atrophy [5,6]. The management of iris cysts depends on whether they are asymptomatic or cause specific complications. It includes observation or intervention, including disruption of the cyst wall with a laser (argon, Nd: YAG), fine-needle aspiration (with or without intracystic injection of absolute alcohol or antimitotic agents), and surgical excision [7,8,9,10].

The final diagnosis is possible through ultrasound biomicroscopic (UBM) examination. This test uses high-frequency ultrasound from 20 MHz to 100 MHz, which allows us to obtain an appropriate resolution of 20–50 µm, with tissue penetration up to 4–7 mm. UBM allows for non-invasive and detailed imaging of the morphology of the iris and the structures behind its posterior surface, especially structures that are not available for visualization in a standard examination using a slit lamp [11,12]. UBM remains the gold standard for the diagnosis of tumors in the anterior segment of the eye [13,14].

Although it may seem that iris cysts have been well studied, in the last dozen or so years, only one original research paper on the clinical characteristics of patients with iris cysts [15] has been published and two review papers were made available online in 2017 [14,16], although both are based on the literature. Other available articles are much older clinical or review papers [3,6,13,17,18,19,20], which were published by the same authors with one exception. Taking this into account, we decided to present a series of 39 patients with iris cysts, analyzing in particular the morphological features and the location of the lesions and the clinical course, including the risk of complications in the long-term follow-up.

## 2. Materials and Methods

This study was performed under approval from the Bioethics Committee of the Medical University of Białystok, in accordance with the ethical standards as laid down in the 1964 Declaration of Helsinki and its later amendments or comparable ethical standards. The subjects provided written, fully informed consent for the examination and use of their clinical data for publication.

We conducted a retrospective review of the charts and electronic images of all adult patients with suspected anterior segment tumors who were examined, treated, and followed at the Department of Ophthalmology, Medical University in Bialystok between April 2016 and February 2020. Sex, age, best-corrected visual acuity (BCVA), intraocular pressure (IOP), slit-lamp evaluation, and images obtained using UBM (Aviso S, v 5.0.0, Quantel Medical, Paris, France) were analyzed.

UBM was performed in all patients by two experienced researchers (J.K., Ł.L.), according to the technique described earlier [21] with a 50 MHz transducer. Images were obtained at the radical meridian conducted through the largest tumor thickness using an eyecup filled with 1% methylcellulose and distilled water. Ultrasound images were assessed for the type of lesion, size, location, echogenicity, external structure (regular/irregular), iris pigment flap eversion, and documented growth. On this basis, 39 patients with iris cysts were identified from the entire group of tumors in the anterior segment of the eye. The dimensions of the cysts were defined as the largest dimension of the base and the greatest dimension of the height, drawn perpendicular to each other, according to the previously described technique [22]. The position of the cysts was assessed in two ways: peripheral, central (at the pupillary margin), and midzonal, and divided into quadrants. The individual quadrants were determined using a clock face, following the rules: (1) upper-nasal quadrant in the right eye: 12.00–3.00, and in the left eye: 9.00–12.00; (2) inferno-nasal quadrant in the right eye: 3.00–6.00, and in the left eye: 6.00–9.00; (3) upper temporal quadrant in the right eye: 9.00–12.00, and in the left eye: 12.00–3.00; and (4) inferno-temporal quadrant in the right eye: 6.00–9.00, and in the left eye: 3.00–6.00. In isolated cases, where the cyst was on the border of two quadrants, it was assigned to the quadrant containing the larger part of it. Multiple cysts were defined as the presence of three or more cysts in one eye or multichamber cysts [6].

Control visits were carried out at 6 month intervals. Basic ophthalmological examination and UBM were performed during each of them. If disturbing symptoms were observed, such as an increase in IOP, an increase in cyst size or deterioration of BCVA, the frequency of visits was higher and adjusted to the local condition. The documented growth of the lesion was assumed to be an increase in its base size or height by ≥20% compared to the previous examination.

## 3. Statistical Analysis

The statistical analysis was performed with the use of the R program, version 3.5.1. The variables studied were presented using descriptive statistics. Nominal variables were compared between the groups using the Fisher’s exact test. The normality of the distribution of quantitative variables was assessed using the Shapiro-Wilk test, indicators of skewness and kurtosis of the data, and visual assessment of histograms. Group comparisons for quantitative data were performed using the nonparametric Mann-Whitney U test or Kruskal-Wallis test. Bonferroni correction was applied for multiple comparisons. The comparative analysis of tumor size with individual studies was performed using the Wilcoxon test for dependent measurements. The correlation of tumor size with selected quantitative parameters was checked using the Spearman correlation index. All tests were two-sided. A *p* value less than 0.05 was considered statistically significant.

## 4. Results

**Figure 1:**
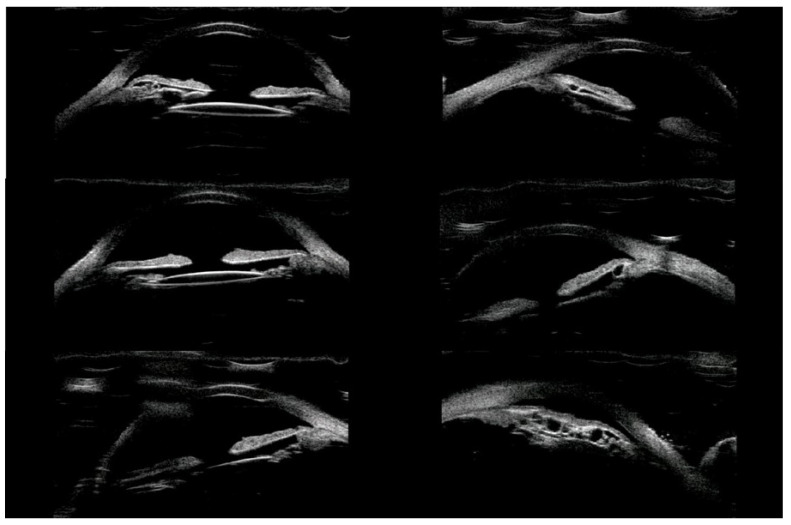
Small cyst of peripheral iris.

**Figure 2:**
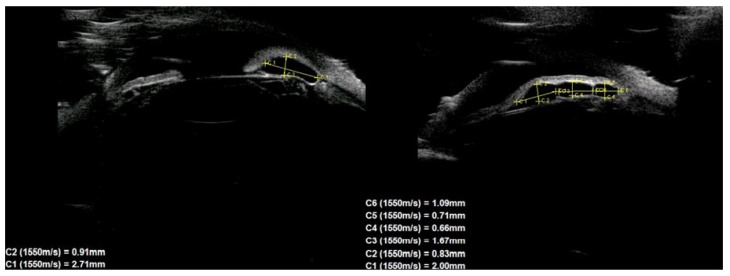
Multichamber cyst.

**Figure 3:**
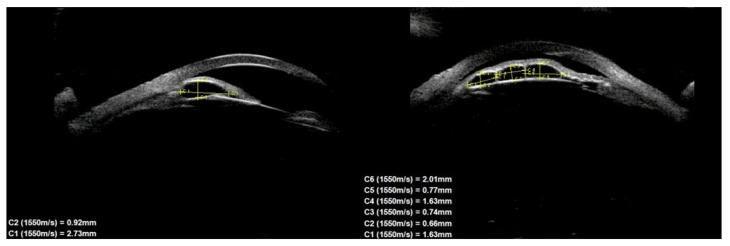
Multichamber cyst.

Of the 39 consecutive patients with iris cysts, 27 (69.2%) were women and 12 (30.8%) were men. The mean age at diagnosis was 40.6 ± 17.48 years ranging from 18 to 84 years. The mean age of men and women did not differ significantly and was 41.1 ± 15.6 and 38.2 ± 18.3 years, respectively, *p* = 0.60. Thirty-four (87.2%) of the cysts were classified as primary and five (12.8%) as secondary. The causes of secondary cysts were as follows: 1—eye injury in childhood, 1—previous cataract surgery using extracapsular cataract extraction, 1—previous phacotrabeculectomy, 2—unknown cause. There were 33 (84.6%) single cysts and 6 (15.4%) multiple cysts (Figure 1, Figure 2 and Figure 3).

**Figure 4:**
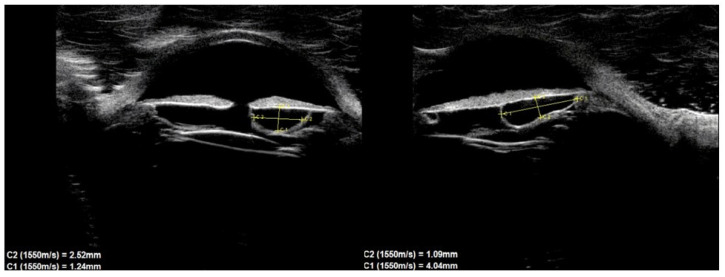
Large cyst covering all zones of iris.

**Figure 5:**
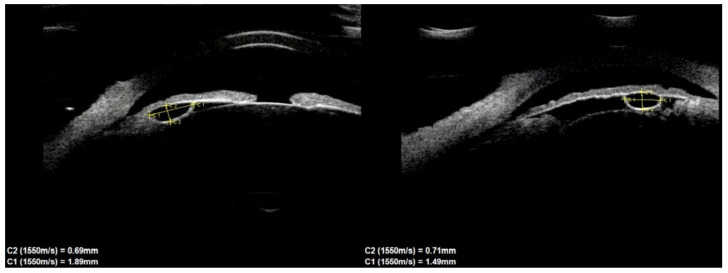
Peripheral cyst.

**Figure 6:**
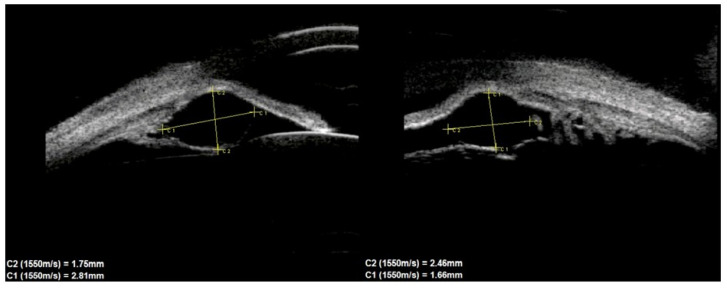
Large peripheral and midzonal cyst.

In terms of location on the iris, 30 (76.9%) cysts were peripheral, 5 (12.8%) were located at the pupillary margin, 2 (5.1%) were midzonal and 2 (5.1%) were multichamber cysts extending from the periphery to the pupillary margin. We did not observe any free-floating cysts. There were 27 primary and 4 secondary cysts peripheral, 4 primary and 1 secondary at the pupillary edge, and 1 midzonal cyst was primary. A total of 23 (59%) cysts were in the lower temporal quadrant, 11 (28.2%) were in the lower nasal quadrant, and 5 (12.8%) were in the upper nasal quadrant. No cysts were found in the superior temporal quadrant. The cyst walls had moderate to high reflectivity in all cases (Figure 4, Figure 5 and Figure 6).

The cyst size measurements based on the UBM test are presented in Table 1. There were no statistically significant differences in cyst size according to sex.

The mean BCVA on the Snellen chart in the group of 39 patients was 0.87 ± 0.25 (median was 1.00, with a range from 0.2 to 1.0). In the group of 34 primary cysts, 22 patients had normal BCVA and 12 patients had reduced BCVA, ranging from 0.5 to 0.9 on the Snellen chart. In the group of secondary cysts, one person had normal visual acuity and four had reduced BCVA, ranging from 0.2 to 0.7 on the Snellen chart. The mean IOP was 15.71 ± 2.78 mmHg (median was 16.0 mmHg, with a range from 10.0 to 24 mmHg).

Cyst-level size was positively correlated with the patient age (rs = 0.38, *p* = 0.003) and negatively correlated with visual acuity (rs = −0.42, *p* = 0.014). The relationship between cyst size and IOP value was not confirmed (Table 2).

Assessment of the anterior segment of the eye with the slit lamp revealed no additional abnormalities such as iris atrophy, sector cataract or anterior uveitis. Elevated IOP were found in three patients with multiple cysts. In all three cases, cysts were located around the periphery of the iris, near the drainage angle. In these patients, perforation of the cyst walls and internal fluid drainage were performed using the Nd: YAG laser according to the previously described technique [23,24]. After the procedure, normalization of IOP was observed in two patients, and in one patient it was necessary to additionally apply local antihypertensive treatment. During follow-up, none of the patients had a cyst growth of more than 20% compared to the first measurement. The mean follow-up length was 26.61 ± 16.13 months, with a range from 24 to 48 months.

## 5. Discussion

In the group of adult patients with iris cysts described in this study, special attention was paid to the characteristics of the cysts, including their location, size, and clinical course over a longer period of observation. Careful knowledge of these clinical features can help to isolate such features that will allow us to conclude with a high degree of probability that we are dealing with a cyst and not a solid tumor. In our study, we diagnosed cysts more often in women than in men (69% vs. 31%), and the average age of the patients at diagnosis was 41.6 years. Köse et al. [15] reported that in a group of cysts, women also predominated (65%), but the mean age of the whole group was lower and amounted to 34.4 years. However, this results from the selection of a group that included both children and adults. A similar selection of subjects was used by Marigo et al. [6]. Nevertheless, the mean age of the patients was 47.7 years, which was higher than that in our study. Moreover, the number of women in this group was slightly higher (54.6%). Based on our research and other studies, it is difficult to determine whether women are more predisposed to the occurrence of cysts or whether they come for prophylactic examination more often [25]. However, there are undoubtedly differences in this respect when comparing cysts with melanomas. Melanomas of the anterior segment of the eye are more common in men, and the mean age at diagnosis is later than that of cysts and is 60 years [2]. The age difference is particularly significant and evident here, considering the fact that especially a pigmented lesion might be easier to notice than a tiny cyst in a routine biomicroscopic examination.

In our study, most cysts were located peripherally (77%) and in the lower quadrants (87%), with a predominance in the lower temporal quadrant (59%). Clearly, peripheral location of cysts is the most prevalent (63–73%) [14,15,16,17]. Peripheral cysts most often occur in people aged 21–40 (75–79%), and least often in seniors >60 years of age (31–38%), and secondly in children (49–61%) [14,16]. As there were no children in our group, and the number of elderly people was also not large, our data seem to correspond with the results of studies by other authors. It can also be assumed that in the case of peripheral cysts, due to their location and the difficulties in their detection, if they concern the iridocilary sulcus, there is a risk of missing such a cyst during routine slit-lamp examination [15,26].

Interesting observations regarding the location of cysts in relation to the quadrants were made. Our group was dominated by cysts located in the lower and temporal parts of the iris. Shields et al. [3] reported that the majority of peripheral cysts, which were dominant in his group, were located temporally (85%), and 73% were located in the lower temporal quadrant. In a group of 37 patients of Köse et al. [15], cysts located at the bottom (38%) and at the temples (32%) also predominated. In turn, the review article of Shield et al. [20], summarizing this topic, indicates that peripheral cysts are often located temporally, but also nasally; the midzonal cysts are located lower with a predominance of the lower temporal quadrant. In the case of the pupillary margin, dislodged and free-floating cysts, the location was random. As in the case of cysts, melanomas of the iris and ciliary body are located in the lower quadrants in 80% of cases [2]. Taking this into account, the location of the cysts cannot be a clinical feature differentiating cysts from iris melanomas.

The pathogenesis of primary iris cysts still remains unclear [13] The majority of cysts arise from splitting of the epithelial layers of the iris, or entrapment of surface ectodermal cells into the iris stroma [3]. Acquired iris cysts most often follow ocular trauma [13]. The implantation of epithelium into the anterior chamber may progress with time and have a higher rate of recurrences and complications [3].

Our assessment of the cyst sizes confirms that these are small lesions with a mean size of 2 × 1.2 mm, and only single multichamber cysts reached a total size of 5.5 × 4 mm. Similar data have been presented by other authors [15,20]. In our study, we found no relationship between cyst size and sex. Compared to cysts, the average sizes of iris and ciliary body melanomas are much larger and amount to 6.5 × 2.7 mm and 11.7 × 6.6 mm, respectively [14]. As reported by Shields et al. [3,20], primary iris cysts rarely progress, with little effect on BCVA and IOP. In most cases, they remain asymptomatic without causing long-term complications—unlike melanomas, which enlarge in size until they become symptomatic. In our study, no cyst growth over the 2–4 year follow-up period was documented. Similar results were reported by Köse et al. [15] and Shields et al. [3]. 

The only complication observed in our group was an increase in IOP. It occurred in three patients, all of whom had multiple cysts located at the drainage angle. IOP normalized after treatment. Multiple cysts are not as rare as previously thought and, in our group, they accounted for 15.4%. Extensive research by Marigo et al. [6], which included a large group of tumors of the anterior segment of the eye, showed that multiple cysts of the iris and ciliary body accounted for 37.8% of cases. Such cysts, due to their larger total size, may cause more complications, although there is currently no convincing research on this subject. In our study, based on six patients, did not allow us to draw any final conclusions in this regard. Studies have described the familial occurrence of iris cysts with autosomal dominant inheritance [16,23]. In these cases, multiple cysts commonly cover more than 1800 perimeters of the filtration angle. In our study group, we had a case of siblings (brother and sister) who developed binocular multiple cysts. Therefore, in the case of multiple cysts, it is worth extending diagnostics to other family members.

We found that the horizontal size of the cysts was positively correlated with the age of the patients and negatively correlated with visual acuity. It can be assumed that the larger dimensions of the base of the cyst in the group of older patients resulted from the fact that these people, due to their age, had these lesions for a very long time and they grew very slowly at that time. As for the correlation of cyst size with visual acuity, the explanation of this relationship requires more research because our analyses did not allow for an unequivocal explanation of this fact.

This study had some limitations that need to be considered. It is a retrospective study, which is important considering the fact that the UBM study is characterized by the variability of the intraobserver and interobserver depending on the experience of the ultrasound examiner [26,27,28,29]. UBM tests in all our patients were performed by two experienced researchers in this field, which guarantees the credibility of our results. In addition, the group of respondents could include a larger number of patients. However, recruiting people with iris cysts is not easy because the vast majority of patients are asymptomatic and do not visit an ophthalmologist. Most iris cysts are detected accidentally during an ophthalmological visit, the reason for which was originally different. The fact that there is little research on this subject only shows how difficult it is to gather a sufficiently large group of patients with iris cysts for analysis. However, the unquestionable advantage of our work may be the homogeneity of the study group and the long observation period.

## 6. Conclusions

Iris cysts are most often located peripherally and in the lower quadrants of the iris, with a predominance in the temporal region. The anatomical location of the cysts cannot be differentiated from solid tumors. There was a positive correlation between the size of the cyst base and age. There were no significant differences in cyst size between men and women. In long-term observation, there is no significant increase in cyst size, which indicates that these changes are benign and safe. The vast majority of cysts are asymptomatic and do not require treatment.

## Figures and Tables

**Table 1:** Cyst size measured with ultrasound biomicroscopy in 39 patients depending on sex.

Sex	Base Width (mm)				Height (mm)			
	*n*	Mean (±SD)	Median (Range)	*p*	*n*	Mean (±SD)	Median (Range)	*p*
The group	39	2.07 ± 0.91	1.87 (1.04–5.64)		39	1.2 ± 0.6	1.09 (0.63–4.04)	
Female	27	2.95 ± 2.34	2.29 (0.96;3.87)	0.512	27	1.40 ± 0.90	1.09 (0.48;2.60)	0.886
Male	12	3.03 ± 2.31	2.41 (1.31;4.33)		12	1.33 ± 0.80	1.21 (0.53;2.93)	

**Table 2:** Correlation between cyst size and age, best corrected visual acuity, and intraocular pressure.

Cyst Size	Base Width		Height	
	Spearman’s Correlation Coefficient r_s_	*p*	Spearman’s Correlation Coefficient r_s_	*p*
Age (years)	0.38	0.003	−0.11	0.390
BCVA	−0.42	0.014	−0.15	0.420
IOP	−0.31	0.113	−0.02	0.939

BCVA—best corrected visual acuity, IOP—intraocular pressure.

## Data Availability

All materials and data are available upon an e-mail request on corresponding author. Names and exact data of the participants of the study may not be available because of privacy policy.
